# Systemic Therapy for Advanced-Stage Hodgkin Lymphoma

**DOI:** 10.3390/cancers18121919

**Published:** 2026-06-12

**Authors:** Varun Iyengar, Kishan Patel, Alison Moskowitz

**Affiliations:** Memorial Sloan Kettering Cancer Center, New York, NY 10065, USA

**Keywords:** classic Hodgkin lymphoma, advanced-stage Hodgkin lymphoma, ABVD, BEACOPP, PET-adapted therapy, brentuximab vedotin, PD-1 blockade, circulating tumor DNA

## Abstract

Advanced-stage classic Hodgkin lymphoma (cHL) represents one of the major success stories in modern oncology, with long-term survival now exceeding 80% for most patients. In this review, we examine the evolution of frontline therapy for advanced-stage cHL, tracing the transition from empiric combination chemotherapy to contemporary, biologically informed treatment strategies. We begin by revisiting the early development of multiagent chemotherapy regimens, including MOPP and ABVD. These regimens established, for the first time, that advanced lymphoma could be cured with systemic therapy. We then discuss efforts to improve outcomes through treatment intensification, which culminated in the development of BEACOPP-based approaches that improved disease control at the cost of substantial acute and long-term toxicity. Subsequently, the incorporation of functional imaging ushered in the era of PET-adapted therapy, enabling dynamic treatment modification based on early response and providing a framework to better balance efficacy with toxicity reduction. Finally, we review the integration of novel agents, including brentuximab vedotin and PD-1 blockade, which have reshaped the frontline treatment landscape and further improved outcomes for high-risk patients while challenging historical chemotherapy paradigms. Collectively, the treatment history of advanced-stage cHL reflects a broader evolution in oncology: from maximizing cytotoxic intensity toward increasingly personalized strategies designed to optimize cure while minimizing long-term harm. Ongoing efforts focused on biomarker-driven risk stratification and the utilization of circulating tumor DNA are poised to further refine this balance in the coming decade.

## Simple Summary

The management of advanced-stage classic Hodgkin lymphoma has undergone a remarkable transformation over the past several decades. Today, most patients can be cured with frontline therapy, a striking achievement that has required decades of therapeutic innovation and careful balancing of treatment efficacy against the risk of both short- and long-term toxicities. As a result, progress in the field has been driven not only by efforts to improve disease control but also by a parallel commitment to reducing treatment-related harm. This review examines the key advances that have shaped modern practice, including the introduction of combination chemotherapy, treatment intensification strategies, response-adapted approaches guided by functional imaging, and the incorporation of targeted and immune-based therapies. Particular attention is given to emerging concepts, such as molecular biomarkers and circulating tumor DNA, that may further personalize treatment selection and help identify patients who are most likely to benefit from treatment escalation or de-escalation in the future.

## 1. Introduction

**Figure 1:**
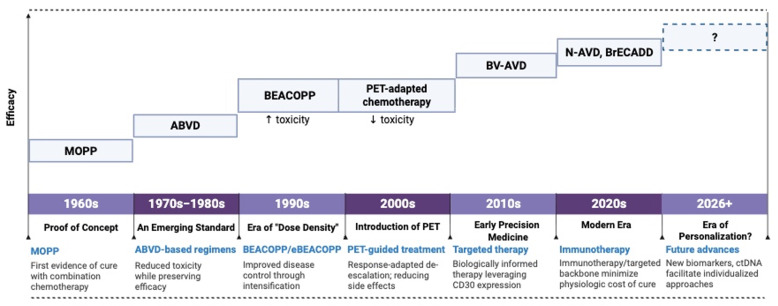
**The Evolution of Advanced-Stage cHL Frontline Therapy.** A chronological progression of standard frontline regimens mapped against approximate survival benchmarks, simplified for the purpose of illustrating the contemporary shift toward high-efficacy, low-toxicity biologic backbones. [Created in BioRender. Varun Iyengar (2026). https://BioRender.com/ (accessed on 5 June 2026)].

Classic Hodgkin lymphoma (cHL) represents one of the definitive success stories of modern oncology. The 5-year survival now approaches 90% across all stages, and even in advanced disease (stages III–IV), long-term survival consistently exceeds 80% [1,2,3]. This success reflects decades of therapeutic innovation, beginning with the development of combination chemotherapy and extending through successive eras of treatment intensification, response-adapted strategies, and, most recently, the incorporation of targeted and immune-based therapies (Figure 1).

Despite these advances, the history of cHL therapy is a narrative of compromise. While early therapeutic breakthroughs established that advanced-stage disease could be “cured” with cytotoxic chemotherapy, these same regimens were associated with significant adverse effects. As a result, the evolution of cHL therapy has been defined not only by efforts to improve cure rates but also by a parallel goal of reducing treatment-related harm.

This review traces the transformation of systemic therapy for advanced-stage cHL, from the empiric intensification of the 20th century to the biologically informed strategies of the modern era. We highlight key therapeutic milestones, discuss current challenges, and explore emerging strategies that aim to personalize therapy while preserving the high cure rates that define this disease.

## 2. Key Biological and Clinical Features

At its biological core, cHL is immunologically complex. The malignancy is defined by a sparse population of tumor cells, derived from germinal-center B-cells and known as Hodgkin and Reed–Sternberg (HRS) cells, that comprise less than 5% of the total tumor mass [4]. These malignant cells orchestrate a reactive tumor microenvironment composed of both immune effectors and stromal cells. The survival of the HRS cell is dependent on this inflammatory milieu, which provides a rich network of cytokine and immune-evasive signaling [5].

Epidemiologically, cHL follows a bimodal age distribution, with a primary peak in young adults (15–30 years) and a secondary peak in those aged 55 and older [1]. In the United States alone, there were an estimated 8720 new diagnoses in 2025, with 1150 deaths attributed to the disease [2]. Histologic subtypes—such as nodular sclerosis, mixed cellularity, lymphocyte-rich, and lymphocyte-depleted variants—are associated with distinct pathologic features but have minimal impact on treatment selection.

Management is guided primarily by disease stage, as described by the Cotswolds-modified Ann Arbor staging system [1]. Advanced stages are defined by the involvement of lymph node regions on both sides of the diaphragm (stage III) or disseminated extranodal involvement (stage IV) and require systemic combination therapy [1,6]. It is important to note that the inclusion of “unfavorable” stage II disease, characterized by factors such as bulky mediastinal masses or B-symptoms, within the advanced-stage category varies by study group and clinical trial protocol. Historically, European groups have included high-risk/unfavorable stage II presentations within their advanced-stage trials (e.g., HD21), whereas many North American studies (e.g., SWOG S1826) have restricted inclusion to stage III and IV disease. It is within all these advanced-stage populations that the balance between maximizing cure and minimizing toxicity is most critical and where therapeutic strategies have seen the most evolution.

## 3. The Development of Combination Chemotherapy

The modern treatment of advanced-stage cHL began with the development of the MOPP regimen (mechlorethamine, vincristine, procarbazine, and prednisone) in the 1960s [7,8]. In a landmark study, DeVita and colleagues showed that six months of MOPP treatment produced unprecedented response rates in advanced disease: in their initial cohort of 43 previously untreated patients, 35 (~80%) achieved a complete response (CR) [7]. With extended follow-up, these responses were shown to be durable, with 68% of complete responders disease-free at 10 years [9]. These seminal findings are widely considered the first evidence that advanced-stage cHL could be cured with chemotherapy.

The success of MOPP, however, was tempered by its toxicity profile. Treatment was associated with myelosuppression and significant effects on fertility, including near-universal azoospermia in men and a high incidence of premature ovarian failure in women [10,11]. Longer-term follow-up revealed an increased risk of secondary malignancies and cardiovascular disease [11,12,13]. These observations introduced a central tension that would shape the next half-century of therapeutic development in cHL: the need to preserve efficacy while reducing treatment-related harm.

In the 1970s, the development of ABVD (Adriamycin, bleomycin, vinblastine, and dacarbazine) at the Milan Cancer Institute offered a solution [14]. Designed to maintain efficacy while minimizing toxicity, ABVD was initially used as a salvage regimen but was quickly evaluated in the frontline setting. Pivotal trials by the Cancer and Leukemia Group B (CALGB) demonstrated that ABVD was superior to MOPP, achieving higher CR rates (82% vs. 67%) and 5-year overall survival (OS) (73% vs. 66%) [15]. Crucially, ABVD achieved these outcomes with a more favorable safety profile—particularly regarding fertility and secondary leukemias—establishing it as the standard of care for advanced-stage disease (Table 1).

## 4. The Era of Intensification

While ABVD represented a significant step forward in the treatment of advanced cHL, it also defined a therapeutic ceiling, and for the approximately 30–40% of patients who experienced primary refractory disease or early relapse, this standard was insufficient [15,17]. Initially, attempts to improve upon ABVD focused on incremental modifications, such as hybrid MOPP/ABVD regimens and the dose-compressed Stanford V program [17,18,19,20]. However, these attempts generally failed to demonstrate consistent improvement and, in most cases, introduced additional toxicity. These failures led to a prevailing belief that meaningfully improving outcomes would require a more radical departure from the ABVD backbone.

The breakthrough in intensification came from the German Hodgkin Study Group (GHSG), who developed the BEACOPP (bleomycin, etoposide, Adriamycin, cyclophosphamide, vincristine, procarbazine, and prednisone) regimen in the 1990s [21]. This protocol was designed to test the Goldie–Coldman hypothesis: that increasing both the dose intensity and the dose density of chemotherapy would maximize tumor cell kill [22]. To test the upper limits of this dose–response relationship, the GHSG evaluated two iterations of the protocol: standard BEACOPP and a more potent, escalated BEACOPP (eBEACOPP), which increased the doses of etoposide, Adriamycin, and cyclophosphamide [16].

The landmark GHSG HD9 trial provided the definitive validation of this dose–response principle [16]. In a pivotal three-arm comparison of eBEACOPP against COPP/ABVD and standard BEACOPP, investigators demonstrated that escalating the intensity of the regimen translated directly into superior clinical outcomes: at five years, freedom-from-treatment-failure (FFTF) rates rose from 69% with COPP/ABVD to 76% for standard BEACOPP to 87% for eBEACOPP (*p* < 0.001 for the comparison with either group). Crucially, eBEACOPP was the only arm to show a survival advantage with a 5-year OS of 91% (vs. 88% for BEACOPP, *p* = 0.06; vs. 83% for COPP/ABVD, *p* = 0.002) [16]. Subsequent randomized studies, including HD2000 [23] and LYSA H34 [24], confirmed that these BEACOPP-based approaches provide superior disease control—though they did not necessarily show the same survival benefit—cementing “dose maximization” as an effective cytotoxic strategy in cHL.

Unfortunately, these gains in efficacy came at the cost of toxicity, echoing the tradeoffs seen in the MOPP era. BEACOPP was associated with high rates of grade 3 and 4 hematologic toxicities, including frequent neutropenia and thrombocytopenia [16,23,24]. Long-term risks again included infertility—nearly universal in men and common in women—as well as an increased incidence of secondary malignancy [25].

Accordingly, this toxicity profile led to a significant geographic divide in clinical practice. In Europe, where the GHSG influence was strongest, eBEACOPP was widely adopted to maximize cure. In the United States, many centers continued to favor ABVD, arguing that the availability of salvage strategies—such as autologous stem cell transplantation (ASCT) [26]—could rescue patients who failed, thereby sparing them the toxicities of upfront intensification.

This persistent debate underscored the limitations of using a uniform approach for all patients. While eBEACOPP improved disease control, it arguably overtreated many patients, while ABVD undertreated a critical minority. These considerations set the stage for the next era of therapy: the development of response-adapted approaches that refined the balance between efficacy and toxicity.

## 5. Response-Adapted Therapy

Part of the resolution to these competing approaches derived not from a novel agent but from a more precise way to risk-stratify patients: the use of positron emission tomography (PET) as an interim assessment of disease response. PET responses have historically been assessed using the five-point Deauville scale, a standardized visual scoring system that compares residual radioactive glucose uptake to physiologic uptake in the mediastinum and liver [27]. In most advanced-stage cHL trials, scores of 1–3 are considered PET-negative and scores of 4–5 PET-positive.

The clinical utility of PET was validated in a landmark study by Gallamini et al., who demonstrated that an interim imaging scan after two cycles of chemotherapy (PET2) was a more powerful predictor of survival than the previously used International Prognostic Score [28]. In their analysis, PET2-negative status was associated with a 2-year PFS of 95%, whereas patients with PET2-positive scans faced a dismal 13% PFS when treatment remained unchanged. In defining these risk cohorts, Gallamini et al. provided an important framework for a personalized approach—namely, intensification for high-risk non-responders and de-escalation for early responders.

### 5.1. Escalation: Rescuing High-Risk Subgroups

In light of the poor outcomes among patients with PET2-positive imaging, several groups became interested in evaluating whether a pivot to more aggressive cytotoxic regimens could salvage these individuals. To this end, the SWOG S0816 trial, in which all patients received two initial cycles of ABVD, provided an important proof of concept [29]. The 82% who achieved PET2-negativity continued ABVD, while the 18% who remained PET2-positive were escalated to six cycles of eBEACOPP. This strategy effectively narrowed the gap in outcomes: at five years, disease-free survival was 76% (95% confidence interval [CI]: 71–81%) for the PET2-negative cohort and 66% (95% CI: 51–76%) for those who escalated [30]—a marked improvement over the historical outcomes reported in the Gallamini era.

The larger GITIL0607 trial further validated this approach in a cohort of 782 patients [31,32]. Following two cycles of ABVD, PET2-negative responders (81%) remained on ABVD, while those with persistent metabolic activity (19%) were escalated to eBEACOPP (with or without rituximab). The trial demonstrated a 3-year PFS of 60% (95% CI: 51–68%) for the PET2-positive group compared to 87% (95% CI: 84–89%) for the PET2-negative group.

Seeking to push the boundaries of intensification even further, the HD0801 trial evaluated the role of ASCT for high-risk patients [33]. Among the 20% of patients who were PET2-positive (defined in this study as Deauville 3–5), treatment was intensified with high-dose chemotherapy and autologous transplant. This approach achieved a 2-year PFS of 74% (95% CI: 62–82%), further validating the potential for high-intensity interventions to salvage patients who demonstrated signs of refractory disease.

Importantly, the interpretation of all these studies does require some caution. Although outcomes among PET2-positive patients treated with intensified approaches compared favorably with historical controls, none of the aforementioned trials randomized patients with positive interim imaging to intensification versus continuation of standard therapy. As a result, the extent to which treatment escalation itself was wholly responsible for the observed improvements in long-term outcomes remains uncertain, although the collective data strongly suggest clinical benefit (Table 2).

### 5.2. De-Escalation: Sparing Toxicity

While intensification sought to improve the ceiling for high-risk patients, de-escalation strategies focused on the majority who achieved early metabolic remission. The goal of these studies was to determine the minimum amount of therapy needed to obtain a cure, mitigating the long-term burden of treatment-related morbidity.

The RATHL trial is widely seen as providing the definitive evidence for de-escalating the ABVD backbone [34]. In this large study of >1200 patients with advanced-stage cHL (including unfavorable stage II disease), 84% were found to be PET2-negative (Deauville 1–3) after two cycles of ABVD and were randomized to either continue ABVD or de-escalate to AVD (omitting bleomycin) for the remaining four cycles. The results established a new paradigm for management: the 3-year PFS was nearly identical between the groups [85.7% (95% CI: 82.1–88.6%) for ABVD vs. 84.4% (95% CI: 80.7–87.5%) for AVD], while the omission of bleomycin significantly reduced the incidence of severe pulmonary toxicity. Long-term follow-up confirmed that this de-escalation strategy did not compromise survival [7-year OS: ABVD, 93.2% (95% CI: 90.2–95.3%) vs. AVD, 93.5% (95% CI: 90.5–95.5%)], establishing PET-adapted AVD as the standard of care in early responders [35].

Other strategies during this era utilized an intensified backbone from the outset, using PET2 to identify candidates for early step-down therapy (Table 2). The AHL2011 trial randomized patients to either standard eBEACOPP or a PET-driven approach [36,37]. In the adaptive arm, patients started with two cycles of eBEACOPP; those who achieved PET2 negativity (87%) were de-escalated to ABVD for the final four cycles. This strategy achieved a 5-year PFS of 85.7% (95% CI: 81.4–89.1%), statistically non-inferior to the 86.2% (95% CI: 81.6–89.8) observed in patients who remained on eBEACOPP for their entire treatment. The GHSG HD18 trial took this a step further by investigating whether the duration of therapy could be truncated [38]. For patients who were PET2-negative after two cycles of eBEACOPP, the trial compared the standard 6–8 cycles against a shortened 4-cycle course, showing similar 5-year PFS across both cohorts [90.8% (95% CI: 87.9–93.7) and 92.2% (95% CI: 89.4–95.0), respectively].

**Table 2:** Key Response-Adapted Clinical Trials.

Trial	Strategy	Initial Therapy	PET2+ Action (Escalation)	PET2− Action (De-Escalation)	Key Outcomes	Takeaway
SWOG S0816 [30]	Escalation: ABVD → eBEACOPP	ABVD × 2	eBEACOPP × 6	ABVD × 4	**PET2+:** 5 yr PFS 66%. **PET2−:** 5 yr PFS 76%.	Escalation to eBEACOPP salvages a high proportion of PET2+ patients compared to historical cohorts.
GITIL 0607 [32]	Escalation: ABVD → eBEACOPP	ABVD × 2	eBEACOPP (± Rituximab)	Continue ABVD	**PET2+:** 3 yr PFS 60%. **PET2−:** 3 yr PFS 87%.	Confirmed that PET-driven intensification improves the ceiling for high-risk patients.
HD0801 [33]	Escalation: ABVD → ASCT	ABVD × 2	Salvage chemotherapy + ASCT	Continue ABVD	**PET2+:** 2 yr PFS 74%. **PET2−:** 2 yr PFS 81%.	Early ASCT is a viable salvage for early non-responders.
RATHL [34,35]	De-escalation: ABVD → AVD	ABVD × 2	Escalated to eBEACOPP	Randomized: ABVD vs. AVD	**PET2−:** 3 yr PFS 85.7% (ABVD) vs. 84.4% (AVD). **7 yr OS:** ~93% for both.	Omission of bleomycin in early responders preserves efficacy and reduces lung toxicity.
AHL2011 [37]	De-escalation: eBEACOPP → ABVD	eBEACOPP × 2	Continue eBEACOPP	ABVD × 4	**PET2−:** 5 yr PFS 85.7% (ABVD) vs. 86.2% (eBEACOPP). **5 yr OS:** ~94% for both.	For early responders, stepping down to ABVD maintains disease control while limiting cumulative toxicity.
HD18 [38]	De-escalation: Truncated eBEACOPP	eBEACOPP × 2	Continue eBEACOPP (×6–8 total cycles)	Reduce to 4 total cycles	**PET2−:** 5 yr PFS 90.8% (6–8 cycles) vs. 92.2% (4 cycles). **5 yr OS:** 95% vs. 98%.	4 cycles of eBEACOPP are sufficient for PET2− patients.

Together, these studies provided important evidence that the cumulative toxicity of intensified chemotherapy—from the risks of pulmonary injury, infertility, and secondary malignancies to the impacts on quality of life [39]—can be reduced through early response-guided treatment modification. They also established that for the vast majority of these patients, the therapeutic benefit can be maintained, thereby setting a new standard in frontline chemotherapy.

## 6. Integration of Targeted Therapy

While PET-adapted strategies optimized the use of cytotoxic agents, they also revealed the inherent limitations of non-specific chemotherapy backbones. Even with response-adapted approaches, an outcome gap persisted between PET2-negative and PET2-positive patients, leading many to believe that further gains would require a shift in therapeutic strategy.

This shift was realized in the early 2000s through the development of brentuximab vedotin (BV). BV is an antibody–drug conjugate (ADC) composed of an anti-CD30 monoclonal antibody linked through a protease-cleavable linker to the microtubule-disrupting agent monomethyl auristatin E (MMAE) [40]. Following binding to CD30-expressing cells, the ADC is internalized, and MMAE is released intracellularly, resulting in selective cytotoxicity while limiting systemic exposure. By leveraging the nearly universal expression of CD30 on HRS cells, the development of BV marked the beginning of a new era in cHL focused on biology-informed therapy.

### Brentuximab Vedotin

The clinical impact of BV in cHL was initially established in the relapsed/refractory (R/R) setting [41,42]. In a landmark study from Younes et al., BV demonstrated an overall response rate (ORR) of 75% (95% CI: 64.9–82.6%) and a CR rate of 34% (95% CI: 25.2–44.4%) in patients who had already failed ASCT [42]. For a population that faced a median OS of less than two years, the observation that BV could induce durable remissions as a single agent was seen as a mandate to move the drug into earlier lines of treatment.

The phase III ECHELON-1 trial was the culmination of this effort and was the first to challenge the long-standing status of ABVD as one of the universal standards of care. By replacing bleomycin with BV (BV-AVD), the study sought to improve disease control in stage III/IV cHL while eliminating the risk of pulmonary toxicity [43]. Initial results demonstrated a modest but statistically significant PFS benefit [82.1% vs. 77.2%; hazard ratio (HR): 0.77; 95% CI: 0.60–0.98; *p* = 0.04], but it was the long-term follow-up that cemented the new regimen’s place in clinical guidelines. BV-AVD showed a survival advantage at six years compared to ABVD (OS: 93.9% vs. 89.4%; HR: 0.59, 95% CI: 0.40–0.88, *p* = 0.009) [44], establishing a new high-water mark for what a frontline regimen could achieve.

Parallel to efforts to improve ABVD, the GHSG also sought to improve upon the eBEACOPP regimen that was popular in Europe. The HD21 trial—which included high-risk stage II cHL, characterized by the presence of B symptoms with either a large mediastinal mass and/or extranodal disease—compared eBEACOPP to BrECADD (BV, etoposide, cyclophosphamide, Adriamycin, dacarbazine, and dexamethasone), replacing the most toxic elements of BEACOPP (procarbazine and vincristine) with BV and simplifying the steroid/chemotherapy schedule [45]. The results were practice-changing: at a median follow-up of four years, BrECADD not only improved PFS (94.3% vs. 90.9%; HR: 0.66, 95% CI: 0.45–0.97, *p* = 0.035) but also had a dramatically better safety profile. Treatment-related morbidity was lower with BrECADD than with eBEACOPP (42% vs. 59%; relative risk: 0.72; 95% CI: 0.65–0.80; *p* < 0.0001), and early markers of fertility recovery were also significantly higher in the BrECADD arm [46].

## 7. The Checkpoint Revolution

While the incorporation of a targeted agent in BV successfully raised the survival ceiling, it was not without its limitations. A significant subset of patients were intolerant to the neurotoxicity of BV, while others continued to relapse, suggesting that there remained opportunities to build on the platform of targeted therapy.

The effort to close this gap coincided with a maturing of our understanding of the cHL tumor microenvironment. Although high expression of PD-L1 in cHL had been previously observed [47,48], it was the more comprehensive characterization of the 9p24.1 genetic locus in the mid-2010s that brought immunotherapy in cHL to the forefront [49]. A landmark paper by Roemer et al. demonstrated that 9p24.1 amplification drives constitutive overexpression of PD-L1 within HRS cells, in turn inhibiting the surrounding T-cell infiltrate and creating an immunosuppressive microenvironment. Around this same time, work by Green et al. described latent Epstein–Barr virus infection—often associated with cHL [50]—as an alternative mechanism for PD-L1 induction, providing yet another pathway for immune evasion [51]. Given that PD-1 inhibitors were simultaneously reaching clinical prominence in solid tumors, their application in cHL became the logical next test [52].

The proof of concept was delivered by the CheckMate 205 [53,54] and KEYNOTE-087 [55,56] trials in R/R cHL. In these studies, PD-1 blockade achieved an ORR of approximately 70% in heavily pre-treated patients, many of whom had already progressed after both ASCT and BV. These results strongly suggested that immunotherapy had the potential to overcome chemoresistance, setting the stage for the evaluation of these agents in earlier lines.

### 7.1. SWOG S1826

The SWOG S1826 trial represented a defining moment in the management of cHL and, in many ways, brought us fully into the modern era. This study aimed to directly compare an immunotherapy-based backbone—nivolumab-AVD (N-AVD)—to the existing standard of care (BV-AVD) for stage III/IV disease [57]. The trial demonstrated a clear and sustained advantage for the checkpoint inhibitor-based regimen; at a median follow-up of 12.1 months, the 1-year PFS for the N-AVD cohort was 94% compared to 86% for BV-AVD [HR 0.48, 95% CI 0.27–0.87, *p* = 0.001]. More recently, 3-year follow-up was presented by Herrera et al. at the 2025 American Society of Hematology Annual Meeting and confirmed the remarkable durability of this approach, demonstrating a sustained PFS advantage for the N-AVD cohort (91% vs. 82% for BV-AVD; HR 0.48, 95% CI 0.34–0.69, *p* < 0.0001) [58].

Equally important, this efficacy was matched by a reassuring safety profile. Rates of febrile neutropenia and sepsis were not increased with N-AVD, and in fact, most adverse events were observed less frequently in that arm. Immune-related adverse events were relatively infrequent with N-AVD and tended to be manageable (Grade 3+ [G3+]: 13.1%; G4: 1.5%; G5: 0%), whereas a higher rate of peripheral neuropathy was seen with BV-AVD (G3+: 8.1% vs. 1.0%). By achieving a 3-year OS of 98% and simultaneously lowering the physiological cost of cure, S1826 seems to have codified a new benchmark for frontline performance. While long-term follow-up remains essential to assess late toxicities and the durability of these remissions, we are hopeful that this high-efficacy, low-toxicity foundation will continue to define the modern era (Table 3).

### 7.2. Older Patients

The shift toward biological backbones has perhaps been most transformative for older and frailer adults (age ≥ 60 years). Historically, these patients have been underrepresented in clinical trials and have faced worse outcomes due to an interplay of more aggressive disease biology and the relative intolerance of intensive chemotherapy [59,60].

However, a dedicated sub-analysis of the S1826 cohort demonstrated that the N-AVD benefit extends to adults ≥ 60 years, offering superior PFS and a more manageable safety profile than BV-AVD [61]. At a median follow-up of 2.1 years, the 2-year PFS was 89% after N-AVD vs. 64% after BV-AVD (HR 0.24, 95% CI: 0.09–0.63, *p* = 0.001). These results have recently been confirmed by phase II data that show high CRR and minimal hematologic stress with frontline N-AVD in elderly patients, even those with geriatric impairments [62]. Together, these data reinforce the robust tolerability of the N-AVD backbone and suggest that the integration of targeted agents may finally offset age as a driver of poor clinical outcomes.

## 8. Future Directions

By consistently breaching the 90% PFS ceiling with improved tolerability, the successes of the S1826 and HD21 trials have begun to resolve the 40-year struggle to balance cure with toxicity. However, these options now introduce a new clinical paradox: when the vast majority of patients achieve a durable frontline remission, the broad application of potent therapy risks overtreating the majority to reach a small subset of non-responders. Therefore, the next frontier in research is to transition to a more precision-based optimization of our therapies, focusing on molecularly guided strategies that align the intensity of therapy with an individual’s risk of relapse.

### 8.1. A Philosophical Divide

The current landscape is defined by a notable philosophical divide. While many centers have adopted N-AVD following the results of S1826, the GHSG has established BrECADD as a formidable alternative. In the absence of head-to-head trials, the selection between these two regimens represents a tradeoff between distinct pros and cons. BrECADD, as validated in the HD21 trial, offers a remarkable 4-year PFS of 94.3%—the highest reported in a randomized trial for advanced disease [45]—but is associated with substantial hematologic toxicity. Conversely, N-AVD trades this acute intensity for the potential of immune toxicities inherent to PD-1 blockade.

In practice, this choice is increasingly dictated by patient-specific factors. Given its robust safety data, the N-AVD backbone offers a more favorable profile for patients ≥ 60 years of age or those with significant comorbidities, where the hematologic stress and high-dose corticosteroids of BrECADD are prohibitive. Conversely, the presence of pre-existing autoimmune conditions or a patient’s reluctance to navigate potential immune-related adverse events can tip the balance toward BrECADD. Notably, while the GHSG has published data regarding fertility preservation with BrECADD [63], emerging evidence suggests that N-AVD carries a similarly low risk of gonadotoxicity [64], neutralizing fertility as a major point of divergence between the two regimens. Ultimately, this divide may only be resolved by long-term follow-up of secondary malignancies and cardiovascular health; in the interim, the hallmark of clinical practice is the use of shared decision-making to align the toxicity profile with a patient’s life priorities.

### 8.2. Redefining Response

Perhaps the most significant open question in the immunotherapy era involves the utility of interim imaging. For over two decades, the Deauville scale has served as the undisputed gatekeeper for treatment escalation and de-escalation. However, the integration of immunotherapy has introduced a disconnect between metabolic activity and clinical outcome. Because checkpoint inhibitors induce a robust inflammatory response, interim PET scans are increasingly prone to false positivity. This was strikingly illustrated in a sub-analysis of the S1826 study, which revealed that 84% of patients who were interim PET-positive remained progression-free at three years on N-AVD [65]. While efforts—such as the COBRA study [66] and an ongoing phase II trial of BV, pembrolizumab, doxorubicin, and dacarbazine [67]—are attempting to maintain PET-adapted de-escalation models, the future of the field likely lies in more objective metrics.

Consequently, research efforts have recently focused on circulating tumor DNA (ctDNA) as a measure of minimal residual disease. By quantifying tumor burden through ultra-sensitive molecular assays, the hope is that we can more reliably distinguish true refractory clones from non-specific metabolic activity. Emerging data have demonstrated that high-throughput sequencing of ctDNA can detect molecular relapses months before they become radiographically apparent [68,69]. A similar approach is being pioneered in the PRECISE-HL trial, which utilizes ctDNA clearance kinetics to guide the reduction in therapy [70]; in this paradigm, patients who achieve rapid clearance may be eligible for de-escalation, while those who demonstrate persistent clones are identified early. In the long run, we believe the future of cHL therapy will leverage these molecularly adapted strategies, setting the stage for a future where treatment intensity is dictated by biological signatures.

### 8.3. The Role of Radiation

Finally, in parallel with the advances in systemic therapy, the role of radiation (RT) in advanced-stage cHL has undergone a transition from a routine component of care to a response-dependent tool. Historically, involved-field RT was utilized to mitigate the risk of relapse in areas of initial bulky disease [71]. However, as the potency of frontline systemic backbones has increased, the efficacy of contemporary agents has rendered routine RT redundant. This was never more evident than in the S1826 trial, where consolidative RT was utilized in less than 1% of the study population [57]. These data suggest that current systemic strategies are sufficient to eradicate the residual clones that previously necessitated local radiation; consequently, the role of RT has been pushed to the periphery of frontline management.

However, the future of RT may not be defined solely by its omission but by its potential to act as a biological adjunct for high-risk patients. Emerging data suggest a synergistic relationship between RT and PD-1 blockade, where localized radiation can potentiate immunotherapy by inducing immunogenic cell death and enhancing antigen presentation [72,73]. With a better understanding of this biology and the advent of molecular tools that can predict resistance to systemic therapy alone, the future will hopefully transform RT into a strategic partner for those whose immune systems require a localized stimulus to potentiate systemic immune activation.

## 9. Conclusions

The evolution of cHL management—progressing from the blunt cytotoxic force of MOPP to the refined biological specificity of N-AVD—stands as one of the defining paradigm shifts in modern oncology. We have successfully raised the curative bar while lowering the physiological cost of survival. As molecular biomarkers, circulating tumor DNA, and response-adapted treatment strategies continue to evolve, the next phase of progress in advanced-stage cHL will be defined by increasingly individualized approaches that maximize cure while minimizing treatment-related toxicity.

## Figures and Tables

**Table 1:** Historical Comparison of Frontline Regimens.

Regimen	Era	Key Components	Landmark Trial	Efficacy *	Major Toxicities
MOPP	1960s	Mechlorethamine, Vincristine, Procarbazine, Prednisone	DeVita et al. [9]	~80% CR	Infertility, Secondary malignancies
ABVD	1970s, 1980s	Adriamycin, Bleomycin, Vinblastine, Dacarbazine	CALGB [15]	82% CR; 73% 5 yr OS	Pulmonary toxicity (Bleomycin), Cardiac (Adriamycin)
eBEACOPP	1990s	Bleomycin, Etoposide, Adriamycin, Cyclophosphamide, Vincristine, Procarbazine, Prednisone	GHSG HD9 [16]	87% 5 yr FFTF; 91% 5 yr OS	Myelosuppression, Infertility, Secondary malignancies

* Because this review spans more than five decades, statistical reporting varies across studies. Hazard ratios and confidence intervals are reported when available; for older trials, efficacy outcomes are presented using the metrics reported in the original publications.

**Table 3:** Modern Frontline Standards.

	N-AVD (S1826) [57]	BrECADD (HD21) [45]
Trial Arms & Duration	N-AVD × 6 cycles vs. BV-AVD × 6 cycles	BrECADD × 4 or 6 cycles * vs. eBEACOPP × 4 or 6 cycles
Inclusion Criteria	Stage III/IV	High-risk stage II + Stage III/IV
Primary Outcome	91% 3-year PFS (vs. 82% BV-AVD)	94.3% 4-year PFS (vs. 90.9% eBEACOPP)
Hazard Ratio	0.48 (95% CI: 0.34–0.69)	0.66 (95% CI: 0.45–0.97)
Overall Survival	98% 3-year OS	98.5% 4-year OS
Grade 3+ Peripheral Neuropathy	1.0% (vs. 8.1% in BV-AVD)	1% (vs. 2% in eBEACOPP)
Other Grade 3+ Toxicities	Immune-Related: 13% (Common: hypothyroidism, rash)	Thrombocytopenia (55%), anemia (30%)

* Number of cycles was PET-guided (4 cycles for PET2-negative, 6 cycles for PET2-positive).

## Data Availability

No new data were created or analyzed in this study. Data sharing is not applicable to this article.
